# Risk Factor Assessment of Lymph Node Metastasis in Patients With FIGO Stage IB1 Cervical Cancer

**DOI:** 10.3389/fonc.2022.809159

**Published:** 2022-03-30

**Authors:** Mu Xu, Xiaoyan Xie, Liangzhi Cai, Yongjin Xie, Qiao Gao, Pengming Sun

**Affiliations:** ^1^ Department of Gynecology, Fujian Maternity and Child Health Hospital, Affiliated Hospital of Fujian Medical University, Fuzhou, China; ^2^ Laboratory of Gynecologic Oncology, Fujian Maternal and Child Health Hospital, Affiliated Hospital of Fujian Medical University, Fuzhou, China

**Keywords:** cervical cancer, lymph node metastasis, lymph node dissection, risk factor, prognosis

## Abstract

**Objectives:**

To assess the risk factors of lymph node metastasis (LNM) in patients with FIGO stage (2009) IB1 cervical cancer (CC).

**Methods:**

Patients with FIGO stage IB1 CC who underwent radical resection between 2012 and 2018 were recruited. The risk factors for LNM were analysed. A recursive partitioning analysis (RPA) was used to divide the patients into risk groups and assess their risk of LNM.

**Results:**

The 5-year overall survival rate was 91.72%, while 80.0% and 93.5% for patients with or without LNM (P<0.05). Multivariable logistic regression analysis showed that lymphovascular invasion (LVI), depth of invasion (DI), tumour size (TS), squamous cell carcinoma (SCC) antigen level were independent risk factors (all P<0.05). Patients were divided into low-risk (no LVI, DI <1/2, TS <2 cm), intermediate-risk (no LVI, DI <1/2, TS ≥2 cm; no LVI, DI ≥1/2, normal SCC level; LVI, DI <1/2, TS <2 cm), and high-risk (no LVI, DI ≥1/2, SCC level ≥1.5 ng/ml; LVI, TS <2 cm, DI ≥1/2; LVI, TS ≥2 cm) groups by RPA according to these four factors. The incidence of LNM among the three groups was 0.00%, 4.40%, and 24.10%, respectively (all P<0.001). The 5-year overall survival rates differed among the groups (98.2%, 92.7%, 83.0%, respectively, P=0.001).

**Conclusions:**

LNM affects the prognosis of patients with FIGO stage IB1 CC. Lymphadenectomy may be avoided for patients in the low-risk group and recommended for those in the high-risk group. Whether dissection is performed in the intermediate-risk group depends on the lymph node biopsy results.

## Introduction

Cervical cancer (CC), the most common gynaecologic malignancy in women worldwide, has the highest incidence and mortality in China and threatens women’s lives and health (1, 2). Lymph node metastasis (LNM) is a critical risk factor for the survival of patients with CC (3, 4). The survival in patients with LNM is obviously worse than that of patients without LNM in the same International Federation of Gynecology and Obstetrics (FIGO) stage. The 5-year overall survival (OS) rate is 90% for early-stage CC without versus less than 50% with LNM (5). According to the 2019 National Comprehensive Cancer Network (NCCN) guideline, radical hysterectomy and pelvic lymph node dissection ± para-aortic lymph node dissection are the standard surgical treatment for FIGO IB1 CC patients without fertility requirements. Fertility-sparing surgery (Radical trachelectomy and pelvic lymph node dissection ± para-aortic lymph node dissection) for stage IB1 has been most validated for tumors ≤2 cm (6). Nevertheless, some patients experience sequelae after lymphadenectomy, such as vascular or nerve injury, pelvic lymphocysts, and lower-limb lymphedema, which can be life-threatening, increase length of hospital stay, and affect quality of life (7–9). The incidence of LNM in early-stage CC is reportedly 15–20% (10, 11) and even lower in FIGO stage IB1 patients at 7–17.4% (12–14). Therefore, exploring the risk factors of LNM in FIGO stage IB1 and classifying patients into different groups to avoid the risk caused by lymph node dissection for low-risk patients is of great clinical significance. Current predictive models for the simultaneous assessment of LNM risk in patients with FIGO stage IB1 CC have not been reported. Therefore, this study explored the available factors for LNM in FIGO stage IB1 CC and stratified the risk of LNM based on recursive partitioning analysis (RPA) to provide a reference for the selection of surgical treatment for early CC.

## Materials and Methods

### Study Population

A total of 284 patients with CC who underwent radical resection at Fujian Provincial Maternity and Children’s Hospital between January 2012 and December 2018 were recruited. The following inclusion criteria were applied: (1) histologically confirmed CC; (2) FIGO stage IB1 with no evidence of tumours invading adjacent organs or distant metastasis; (3) having undergone radical hysterectomy with pelvic lymphadenectomy; and (4) patients had completed fertility or without fertility requirements. The risk of surgery had been fully informed and all patients signed informed consent before operation and required radical hysterectomy. The study excluded patients who had distant metastases in the liver, lung, or peritoneum/pelvic cavity diagnosed before or during the surgery, those who underwent preoperative neoadjuvant radiotherapy and/or chemotherapy, or those whose medical records were incomplete/inaccurate. FIGO staging criteria (2009) were used for tumour staging. According to the postoperative pathological examination results, supplementary chemotherapy, radiotherapy, or concurrent chemoradiotherapy should be performed in cases of high-risk factors such as worse pathological differentiation degree, positive pelvic LNM, parametrial involvement, deep muscle layer infiltration, positive lymphovascular invasion (LVI), or positive surgical resection margins.

### Follow-Up Investigation

A postoperative follow-up assessment was performed every 3 months for 2 years and then every 6 months during years 3–5. Most routine follow-up appointments included a physical examination, vaginal examination, laboratory testing (including cancer antigen 125 (Ca125) and squamous cell carcinoma (SCC) antigen), chest radiography, and pelvic ultrasonography. Lung computed tomography or pelvic magnetic resonance imaging was performed when necessary. OS was defined as the time from surgery to death of any cause or to the time of censoring on the date of the last follow-up. The final follow-up evaluation was conducted in December 2020. The median follow-up period was 54.3 (range, 6.4–97.1) months.

### Statistical Analysis

The chi-square test or Fisher’s exact probability method was used to compare the classified variables. A t-test or the Mann-Whitney U test was used to analyse differences in numerical variables. Survival curves were constructed according to the Kaplan–Meier method, and differences between curves were analysed using the log-rank test. Variables with values of P<0.05 on univariate analysis were subjected to a multivariate logistic regression analysis. According to those results, recursive partitioning analysis (RPA) was used to divide the patients into different risk groups. The groups with a similar incidence of LNM were reintegrated into a single risk group. Finally, the low-risk, intermediate-risk, and high-risk groups were determined with the incidence of LNM increasing in turn. The statistical analyses were performed using SPSS for Windows version 26.0 (SPSS Inc., Chicago, IL, USA) and R x64 ver. 4.1.0 (R Foundation for Statistical Computing, Vienna, Austria). All tests were two-sided, and statistical significance was determined at values of P<0.05.

## Results

### Clinicopathological Characteristics

A total of 284 patients were included in the study. Their clinicopathological features are presented in **Table 1**. The incidence of LNM was 8.4%. The median patient age was 46 (range, 24–66) years. A body mass index (BMI) of <24 and ≥24 was observed in 188 (66.2%) and 96 (33.8%) cases, respectively. Squamous, adenoma, and adenosquamous carcinomas were observed in 214 (75.4%), 59 (20.8%), and 11 (3.9%) cases, respectively. Invasion depths of <1/2 and ≥ 1/2 of the stroma were observed in 168 (59.2%) and 116 (40.8%) cases, respectively. There were 77 (27.1%) and 207 (72.9%) cases of positive LVI versus no LVI, respectively. There were 178 (62.7%) and 106 (37.3%) patients with a tumour size (TS) <2 cm and ≥2 cm, respectively. There were 163 (57.4%) and 121 (42.6%) cases with a normal or high Ca125 level, respectively. There were 187 (65.8%) and 97 (34.2%) cases of a normal and high SCC level, respectively (**Table 1**).

**Table 1:** Patient Demographics and Clinical Characteristics of CC Patients by LNM.

Parameters	Total	LNM		P value
	(n=284)	Absent (n=260)	Present (n=24)	
	n (%)	n (%)	n (%)	
**Age (y)**				0.088
<45	119 (41.9)	105 (40.4)	14 (58.3)	
≥45	165 (58.1)	155 (59.6)	10 (41.7)	
**BMI (kg/m^2^)**				0.341
<24	188 (66.2)	170 (65.4)	18 (75.0)	
≥24	96 (33.8)	90 (34.6)	6 (25.0)	
**Surgery**				0.829
Open	148 (52.1)	136 (52.3)	12 (50.0)	
Laparoscopy	136 (47.9)	124 (47.7)	12 (50.0)	
**Histological type**				0.069
Squamous	214 (75.4)	193 (74.2)	21 (87.5)	
Adenocarcinoma	59 (20.8)	58 (22.3)	1 (4.2)	
Adenosquamous	11 (3.9)	9 (3.5)	2 (8.3)	
**Depth of invasion**				<0.001
<1/2	168 (59.2)	165 (63.5)	3 (12.5)	
≥1/2	116 (40.8)	95 (36.5)	21 (87.5)	
**Lymphovascular invasion**				<0.001
No	207 (72.9)	200 (76.9)	7 (29.2)	
Yes	77 (27.1)	60 (23.1)	17 (70.8)	
**Tumor Size (cm)**				<0.001
<2	178 (62.7)	172 (66.2)	6 (25.0)	
≥2	106 (37.3)	88 (33.8)	18 (75.0)	
**Ca125 (U/ml)**				0.039
<35	163 (57.4)	154 (59.2)	9 (37.5)	
≥35	121 (42.6)	106 (40.8)	15 (62.5)	
**SCC (ng/ml)**				<0.001
<1.5	187 (65.8)	180 (69.2)	7 (29.2)	
≥1.5	97 (34.2)	80 (30.8)	17 (70.8)	

CC, cervical cancer; LNM, lymph node metastasis; BMI, body mass index; Ca125, cancer antigen 125; SCC, squamous cell carcinoma antigen.

### Impact of LNM on Survival Outcomes

**Figure 1:**
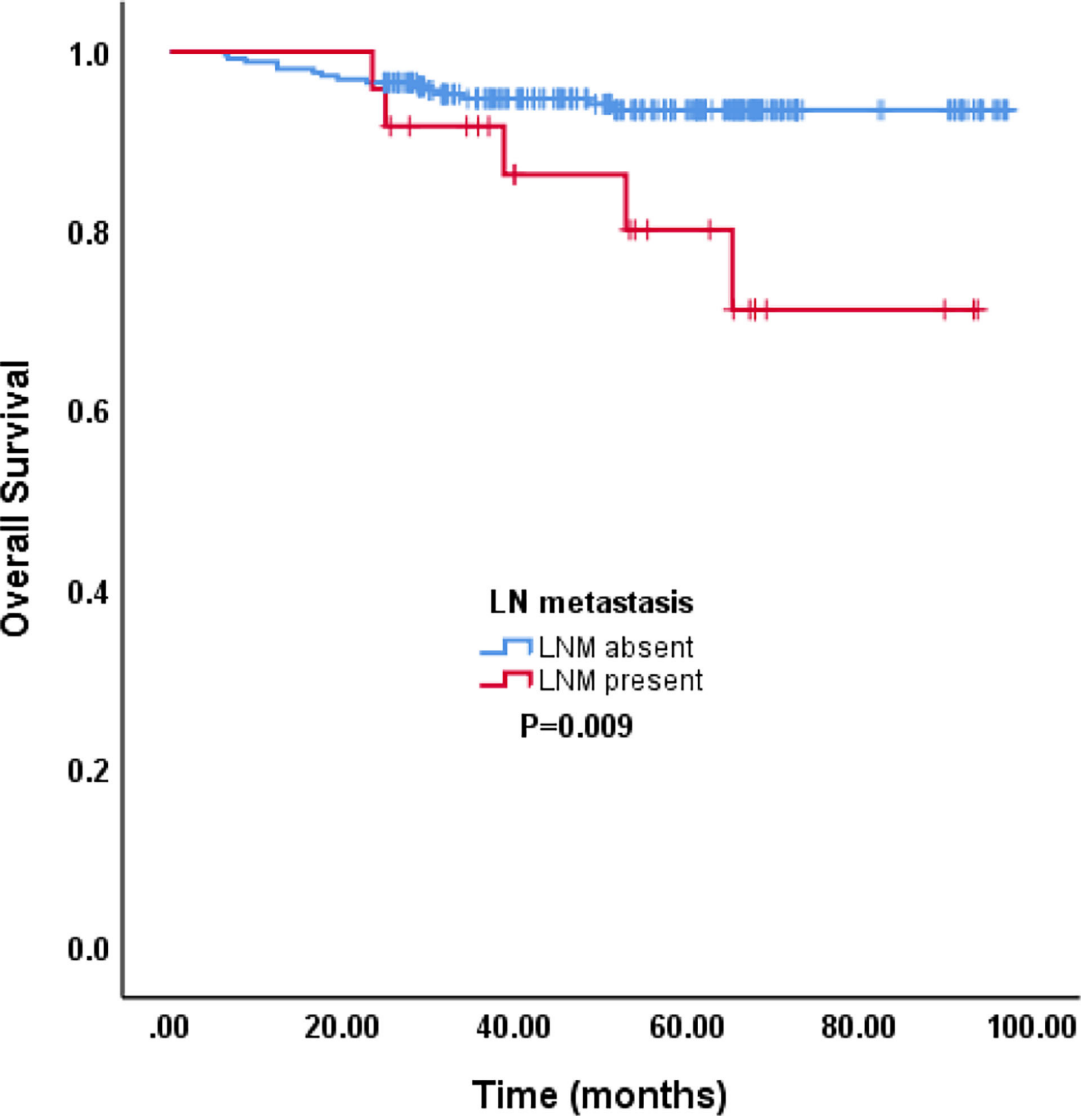
The survival between LNM absent and LNM present.

The median follow-up time was 54.3 (range, 6.4–97.1) months. The 5-year OS rate of all patients and in those with and without LNM were 91.72%, 80.0%, and 93.5%, respectively (P=0.009) (**Figure 1**).

### Risk Factor Analysis of LNM

The univariate analysis showed that TS, depth of invasion (DI), LVI, SCC antigen level, and Ca125 level were associated with LNM (all P<0.05) (**Table 1**). In the multivariate logistic regression analysis, TS (odds ratio (OR), 0.351; 95% confidence interval (CI), 0.123–0.992; P=0.044), DI (OR, 0.205; 95% CI, 0.054–0.772; P=0.019), LVI (OR, 0.281; 95% CI, 0.100–0.785; P=0.015), SCC antigen level (OR, 0.338; 95% CI, 0.123–0.924; P=0.035) were independent factors for LNM (**Table 2**).

**Table 2:** Multivariable Binary Logistic Regression Analysis of CC for the LNM.

	Multivariable Analysis			
	Odds Ratio	95%CI		P
**Tumor Size (cm)**				0.044
<2	Ref			
≥2	0.351	0.123	0.992	
**Depth of invasion**				0.019
<1/2	Ref			
≥1/2	0.205	0.054	0.772	
**Lymphovascular invasion**				0.015
No	Ref			
Yes	0.281	0.100	0.785	
**Ca125(U/ml)**				0.189
<35	Ref			
≥35	0.523	0.199	1.375	
**SCC(ng/ml)**				0.035
<1.5	Ref			
≥1.5	0.338	0.123	0.924	

CC, cervical cancer; LNM, lymph node metastasis; SCC, squamous cell carcinoma.

### Risk Groups of LNM by RPA

**Figure 2:**
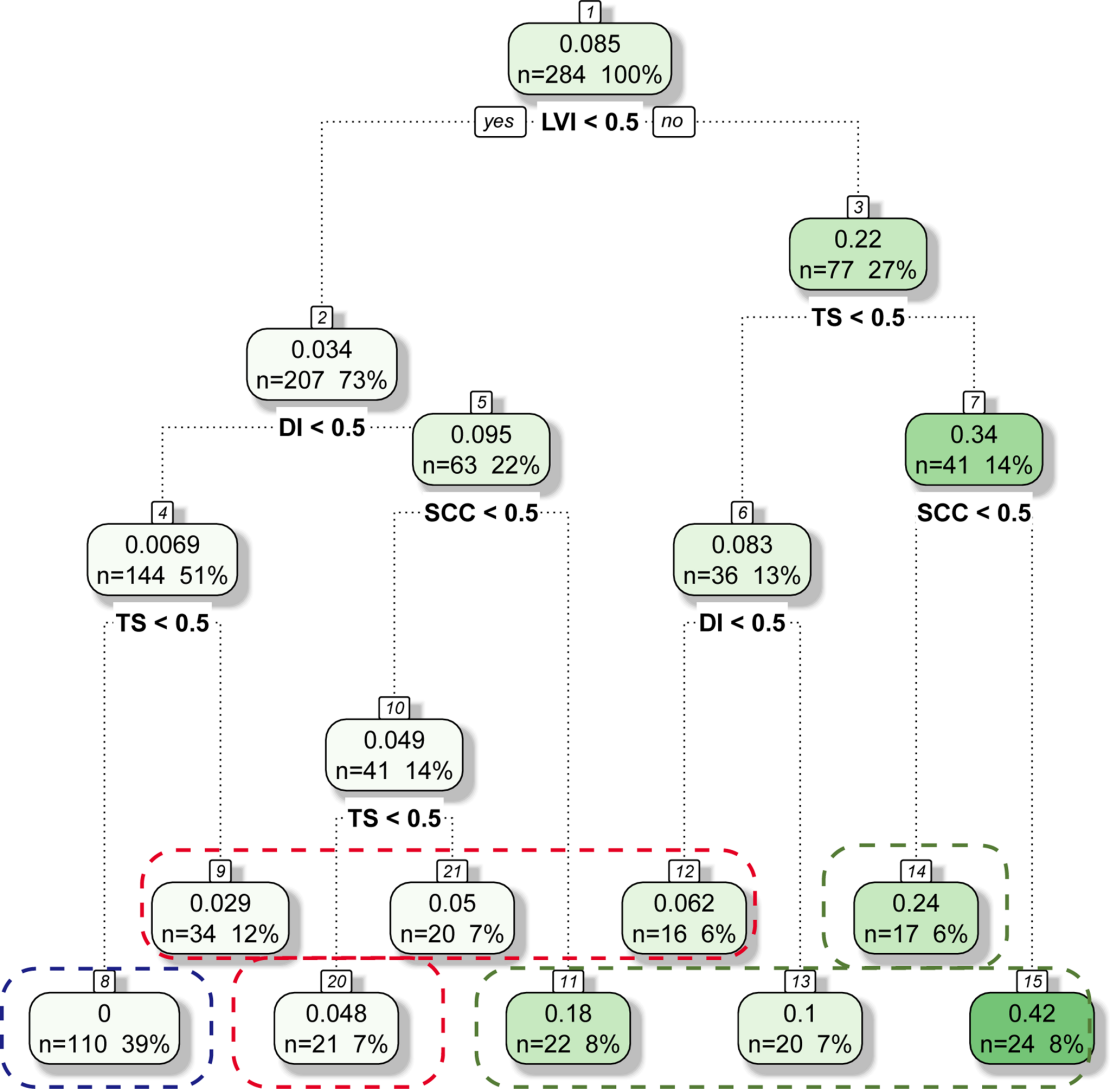
Classification Tree for LNM Status. Blue line, Red line, Green line represents Low-Risk Group, Intermediate-Risk Group and High-Risk Group, respectively.

Based on the results of the multivariable analysis, RPA using the four independent risk factors was performed to classify the patients into different risk groups. The group was divided into subgroups according to the R software prioritisation of the binary variables. Finally, the patients were reclassified into nine groups. Patients with a similar incidence of LNM were merged. The patients were ultimately divided into low-risk, intermediate-risk, and high-risk groups (**Figure 2**). In the model, there were 110 low-risk patients (38.7%) (no LVI, DI <1/2, TS <2 cm), 91 intermediate-risk patients (32.1%) (no LVI, DI <1/2, TS ≥2 cm; no LVI, DI ≥1/2, normal SCC antigen level; LVI, DI <1/2, TS <2 cm), and 83 high-risk patients (29.2%) (no LVI, DI ≥1/2, SCC antigen level ≥1.5 ng/mL; LVI, TS <2 cm, DI ≥1/2; LVI, TS ≥2 cm).

### Difference in LNM Rate and Prognosis by RPA Findings

**Figure 3:**
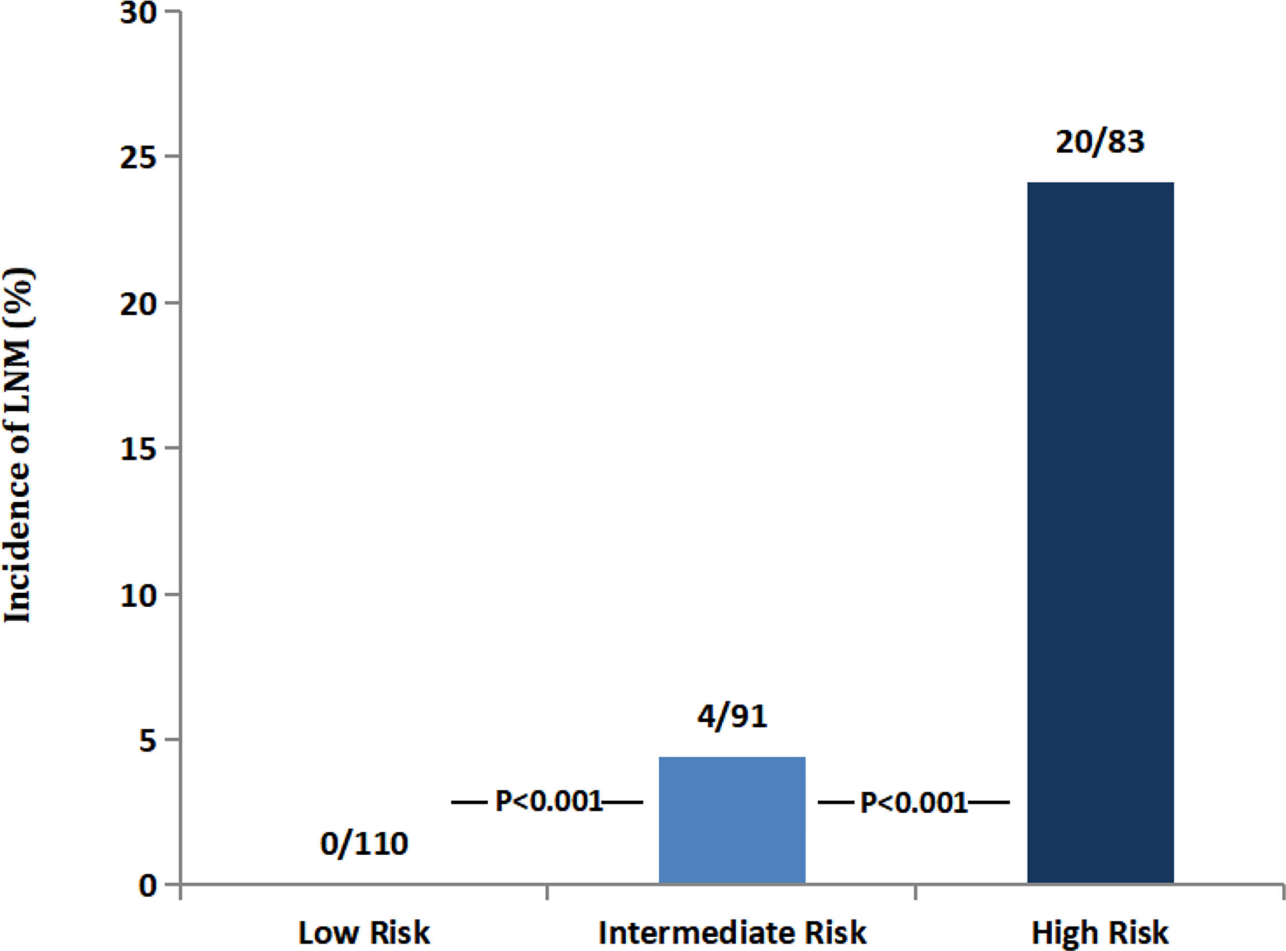
The number of LNM according to RPA risk stratifications.

**Figure 4:**
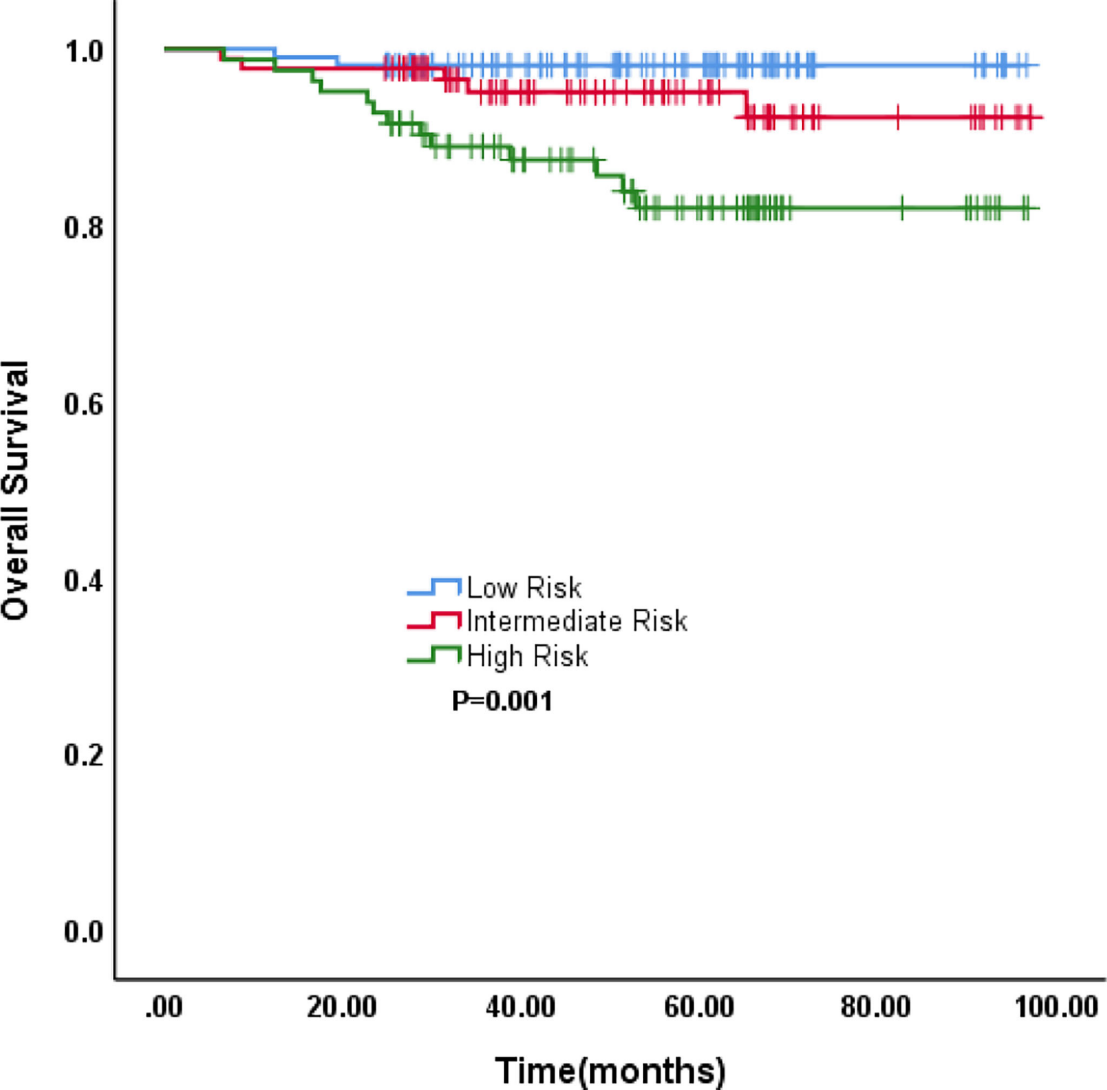
The survival according to RPA risk stratifications.

The incidence of LNM was 0.00%, 4.40%, and 24.10% in the low-risk, intermediate-risk, and high-risk groups, respectively (all P<0.001; **Figure 3**). The 5-year OS rates were 98.2%, 92.7%, and 82.0%, respectively, which were also significantly different (P=0.001; **Figure 4**).

## Discussion

CC is a common gynaecologic malignant tumour for which postoperative recurrence and metastasis are the main causes of death (15, 16). Patients with early-stage CC always have a better prognosis after surgery (2) but worse prognosis when proven to be metastatic. LNM, as the main mode of metastasis in patients with CC, is a high-risk factor for recurrence and greatly impacts treatment and prognosis (3, 4). LNM was also formally included in the FIGO staging system in 2018 (17). Therefore, pelvic lymph node dissection is important. According to the NCCN, the standard surgical treatment is radical hysterectomy and pelvic lymphadenectomy in patients with FIGO stage IB1 CC without fertility requirements (6). However, some patients experience sequelae from this procedure (such as vascular or nerve injury, pelvic lymphocyst, and lower-limb lymphedema), which may increase the length of hospital stay and affect quality of life (7–9). In addition, the rate of LNM in FIGO stage IB1 patients was 7–17.4% (12–14). Lymphadenectomy may not have a survival benefit, but it may increase the incidence of postoperative complications in LNM-negative patients. LNM-positive patients not treated with lymphadenectomy may experience recurrence within a short period postoperative. Therefore, it is of great significance to explore the risk factors influencing LNM and conduct a risk assessment in patients with FIGO stage IB1 CC.

This study showed an 8.4% (24/284) rate of LNM in patients with FIGO stage IB1 CC, a finding that is in accordance with those of other studies (12–14). The 5-year OS rate in patients with LNM was significant lower than patients without LNM. As the patients were selected from January 2012 to December 2018 before the results of LACC trails (18), a certain proportion of patients underwent radical hysterectomy *via* laparoscopy. However, the results showed no prognostic differences between patients *via* laparoscopy or not (**Supplementary Figure 1**). This result needed further confirmation in future studies. The rate of LNM also showed no significant difference between the open and laparoscopic surgery in the study. In addition, with the development of technology, robotic surgery has been gradually applied in clinical. Previous studies showed the equivalence of robotic and laparoscopic approaches to radical surgery of early CC patients (19), so did the salvage lymphadenectomy (20). Therefore, it also may be a good choice in clinical practice in the future.

The multivariable logistic regression analysis showed that LVI, DI, TS, and SCC antigen level were independent risk factors, which is also consistent with previous reports (4, 21–23). LVI is closely associated with LNM in CC. Invasion into the space between lymphatic endothelial cells is an indispensable step for the metastasis of cancer cells; thus, it often predicts a poor prognosis. The larger the tumour diameter and the deeper the musculature invasion, the more likely tumour cells will invade the intravascular system, and thus the more likely the development of LNM. The SCC antigen is a specific serum tumour marker for CC that was first discovered by Kato in 1977 (24). The higher the SCC antigen level, the more aggressive the tumour and the higher the probability of LNM (25, 26). However, previous studies (4, 21) analysed only the independent risk factors affecting LNM without further risk grouping, resulting in some limitations in determining whether lymphadenectomy should also be performed. This study was the first to report a risk stratification by RPA based on these factors, and it provides a reference for whether lymphadenectomy should be performed simultaneously in patients with early-stage CC.

RPA is a statistical method for multivariable analyses that divides groups into subgroups according to the priority of several binary independent variables to correctly classify the members of a group. As a result, a concise decision tree is generated intuitively to determine decision rules with higher sensitivity and specificity (27). This method is widely used in medical decision-making. The RPA was first used by Goldman to establish a decision tree for the diagnosis of patients with acute chest pain in 1982 (28). It was also used to group patients with acute decompensated heart failure by Fonarow (29). In this study, the rate of LNM was 0.00% in patients with no LVI, a DI <1/2, and a TS <2 cm (low-risk group) according to the RPA. The negative LVI, less tumor size and less depth of invasion meant the less invasion in the intravascular system and parametrial involvement, leading to the less possibility of LNM (21–23, 30). Lymphadenectomy may be avoided in these patients to reduce postoperative complications. The LNM rate was as high as 24.10% in high-risk patients, including: those with no LVI, a DI ≥ 1/2, and an SCC antigen level ≥1.5 ng/mL; those with LVI, a TS <2 cm, and a DI ≥1/2; and those with LVI and a TS ≥2 cm. Therefore, simultaneous lymphadenectomy is recommended.

Sentinel lymph node biopsy (SLNB) has recently been used in patients with early-stage CC. Compared to pelvic lymph node dissection, SLNB reduces the incidence of postoperative complications and improves quality of life without affecting the survival prognosis (31–34). SLNB could be applied in the intermediate-risk group (no LVI, DI <1/2, TS ≥2 cm; no LVI, DI ≥1/2, normal SCC level; LVI, DI <1/2, TS <2 cm) as the rate of LNM was 4.40%. Whether to perform simultaneous lymphadenectomy could be determined based on the SLNB results. In addition, the LNM rate differed significantly between the three groups here and also on the survival analysis, indicating that risk grouping based on these four risk factors has a certain reference significance in clinical decision-making.

This study had several limitations. First, it was retrospective in design, which inevitably involves data selection bias. Second, the research was conducted in a single centre with small case numbers, and our findings require further confirmation in prospective multicentre studies. However, to our knowledge, this study is the first to explore the risk factors affecting LNM in patients with FIGO stage IB1 CC. Furthermore, the RPA was used to classify the risk groups to make the model more clinically useful. Based on these findings, we have proposed recommendations for lymphadenectomy for different risk groups with early CC, which will aid surgeons make better clinical decisions, showing the important clinical significance of our study.

## Data Availability Statement

The raw data supporting the conclusions of this article will be made available by the authors, without undue reservation.

## Ethics Statement

The present study was approved by the Ethics Committee of the Fujian Provincial Maternity and Children’s Hospital. Written informed consent was obtained from all participants. The patients/participants provided their written informed consent to participate in this study.

## Author Contributions

MX and XX designed, conceived this study, and wrote the paper. YX and QG contributed to the literature search and collect the data. LC was involved in data extraction and analyzed the data. PS revised the paper. All authors have approved the final edition of the manuscript.

## Funding

This study was funded by Fujian Provincial Maternity and Children’s Hospital Natural Science Foundation (Grant No. YCXM 20–03).

## Conflict of Interest

The authors declare that the research was conducted in the absence of any commercial or financial relationships that could be construed as a potential conflict of interest.

## Publisher’s Note

All claims expressed in this article are solely those of the authors and do not necessarily represent those of their affiliated organizations, or those of the publisher, the editors and the reviewers. Any product that may be evaluated in this article, or claim that may be made by its manufacturer, is not guaranteed or endorsed by the publisher.

## Supplementary Material

Supplementary Figure 1 The survival between laparoscopic surgery and open surgery.
