# Supplementary figures and images for: Risk Factor Assessment of Lymph Node Metastasis in Patients With FIGO Stage IB1 Cervical Cancer
Source: Front Oncol. 2022 Mar 30;12:809159. doi: 10.3389/fonc.2022.809159 (PMC9007329; doi:10.3389/fonc.2022.809159)

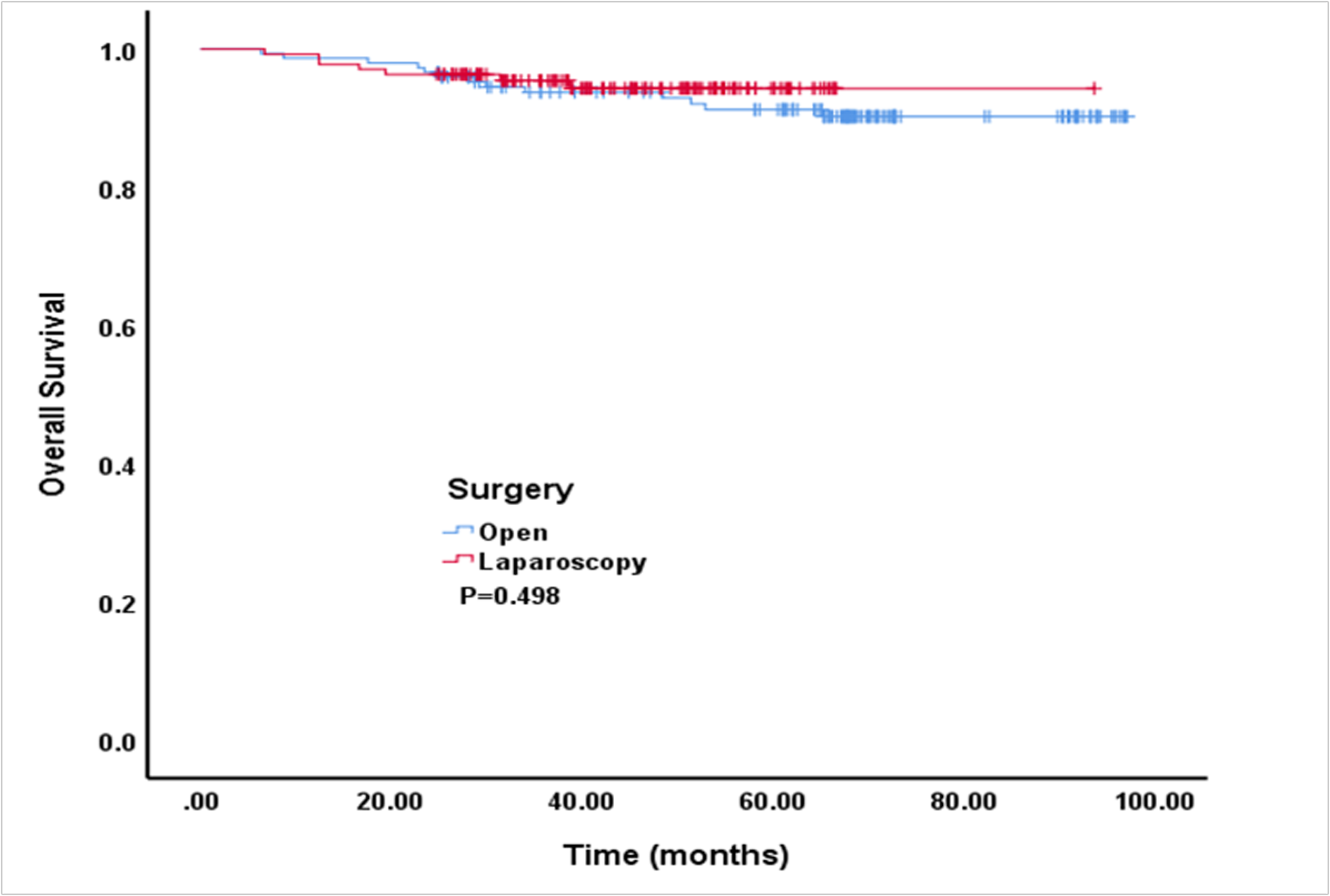

Supplement: Supplementary Figure 1 — The survival between laparoscopic surgery and open surgery. [file Image_1.tif]
